# Cocaine—Its Use and Abuse

**Published:** 1897-05

**Authors:** 


### Cocaine—Its Use and Abuse.

Cocaine is usually employed in solution, and this should always be fresh. Solutions that are kept for several days gradually decompose, a flocculent deposit forms, and the strength of the cocaine is weakened. It is easy to observe the difference in anæsthetic effect between two equally strong solutions of cocaine, one of which is fresh, and the other, one or two weeks old. It does not seem practicable to preserve the solution in good condition by the addition of boracic acid, or any other antiseptic.

The strength of the solution should be governed by the nature of the case in which it is to be used. If the solution is to be injected hypodermically, or is to be applied to an extensive area of absorbent surface, as in the nose or throat, the solution should be weak, i. e., 2 to 3 percent, because under such conditions the opportunities for the absorption of the cocaine into the general circulation are the best, and if strong solutions are employed, toxic symptoms will most probably develop. Likewise, when applied to parts in which the circulation can not be controlled.

On the other hand, when the cocaine is to be applied to only a limited area of an absorbent surface, as for the removal of a spur of the nasal septum, or the application of the cautery to a turbinated body, the solution should be strong, i. e., 5 to 10 percent. Also when used on parts where the circulation, by use of the tourniquet, can be controlled, the solution may be strong. Under such conditions the gradual loosening of the tourniquet after the operation will enable the hemorrhage to wash the cocaine out of the tissues, and as but little of the drug enters the general circulation toxic symptoms rarely result.

The methods of application will next be considered. Mucous, ulcerated and denuded surfaces absorb cocaine, but the healthy skin does not. When mucous or sub-mucous tissues are to be anæsthetized the solution is applied directly to the membrane, and is readily absorbed. The same may be said of ulcerated, raw or abraded surfaces. An exception in the case of mucous membranes is found when they are violently inflamed. One can not successfully anæsthetize a conjunctiva, the seat of an acute purulent inflammation. Under such conditions the cocaine seems either not to be absorbed, or, if absorbed, is too rapidly removed by the increased circulation to produce its local effect.

It may be stated here that cocaine is absorbed with especial freedom by wounds, where there is no present hemorrhage to wash the drug away. A very small amount of cocaine applied to such wounds is often followed by grave toxic symptoms. If the skin or subcutaneous tissues are to be cocainized the solution should be injected hypodermically. Or galvanism may be used to drive the solution through the skin. If the sponge or cotton attached to the positive pole of a galvanic battery be moistened with a solution of cocaine, and applied to the skin, anæsthesia of the parts results in from five to fifteen minutes, depending on the strength of the solution and of the galvanic current.

The length of time necessary for the production of cocaine anæsthesia varies. When applied to a mucous surface, as the nose, the full effect is obtained in from five to ten minutes. When galvanism is used to drive the cocaine through the skin, ten to fifteen minutes are necessary. But when injected into the tissues, anæsthesia is developed within one minute. The degree of anæsthesia varies with the strength of the solution and the duration of the application.

The method by which cocaine produces anæsthesia is not known. The theory that it is due to an anæmia of the tissues, caused by the stringent action of the cocaine upon the blood vessels, is clearly erroneous. Cocaine produces anæsthesia only when it mingles in solution with the interstitial fluids of the part; and then the anesthesia is the result of the peculiar action of the cocaine upon the nerve-fibers, and can not be further explained.

The duration of the anæsthesia is important. It will last practically as long as the cocaine is kept confined in the tissues. Cocaine is removed from the tissues by the blood; and is either thrown off externally, as with the blood in hemorrhage, or returns with the blood to mingle with the general circulation.

It follows, then, that if a tourniquet be applied to a part, as a finger, so as to arrest circulation, and the cocaine is then injected, that the anæsthesia will last while the tourniquet is kept on. It also follows that where no tourniquet is applied the anæsthesia will last *cæteris paribus* in inverse proportion to the vascularity of the part. For this reason the anæsthesia lasts longer in non-vascular tissue, as the cornea, than it does in a vascular one, as the nasal mucous membrane.

In operating on vascular parts the hemorrhage, unless controlled, washes out the cocaine and sensibility speedily returns. It is, therefore, important to complete the operation as speedily as possible; and the more so since anæsthesia can not be re-established by applying more cocaine to a bleeding surface from which it is constantly washed.

Following cocaine anæsthesia the return of sensibility is often marked by a stinging, aching sensation in the part, which may, in fact, last for hours and cause decided suffering. I have often noticed that this afterpain is in proportion to the amount of cocaine used. For its alleviation we should not commit the error of applying more cocaine, for this would only prolong and increase the trouble.

It has been noticed that wounds made under cocaine anæsthesia often fail to unite by primary union. So frequently has this been observed that an explanation has been sought. Two causes are assigned: One is that cocaine, by its effect on the tissues, so impairs their vitality as to defeat union by "first intention." The second is, that it is not the cocaine *per se*, but the septic quality of the solution used that prevents primary union. The solution itself may be septic, or, if injected, the syringe may be septic, and thus the wound is infected. In a word, that it is sepsis and not cocaine that prevents primary union. That this latter explanation is the correct one I do not doubt. For, if a fresh aseptic solution be injected with a thoroughly aseptic syringe, primary union is as easily obtained as it is in the same wounds made under general anæsthesia. The syringe to be used should always be cleansed with a carbolic solution.

Toxic symptoms produced by cocaine will next be considered. These symptoms are more dependent upon the susceptibility of the individual than upon the amount of the cocaine used, or the method of its employment. Nor are these susceptible persons confined to frail or debilitated classes. On the contrary, one as often meets with cocaine poisoning in strong, robust men as among others. Some patients are so sensitive to cocaine that the application of a few drops of a weak solution to any absorbent surface is rapidly followed by the gravest symptoms. Recently I applied to the everted eyelid of a robust young man a few drops of a cocaine solution for the incision of a chalazion, and had a marked case of poisoning within three minutes. Such cases might be multiplied.

Nor is it possible by any known means to judge of the susceptibility of a patient until the cocaine has been used. For this reason the first use of cocaine in an individual should be guarded until we are sure there is no idiosyncrasy present.

The toxic symptoms are a marked loquacity, a feeling of weakness, tingling sensation in the extremities, general nervousness, blanching of the skin, cold perspiration, rapid feeble pulse, superficial respiration, vomiting, dilated pupils, cyanosis, unconsciousness and convulsions. Ordinarily the lighter cases rapidly recover, and leave no bad results, but the graver ones may increase and end in death by arrest of respiration.

These symptoms are best combatted by laying the patient down, giving whisky, either by the mouth or hypodermically; hypodermatic strychnine or atropine, and artificial respiration.

Last, but not least, is the cocaine habit. In many people the effect of cocain is so pleasant, the stimulating effect, both physical and mental, is so agreeable, and the relief from certain local conditions following its use is so gratifying that the patient insists on its continued use. And when, after a time, the physician declines to employ it further, the patient goes, without his physician's knowledge, to the drug-store and obtains and uses it himself. Thus is not unfrequently established the cocaine habit.

Those who are given to the alcoholic or opium habit seem predisposed to the cocaine habit, and in such patients cocaine should be used sparingly. As an additional precaution against this habit the patient, when practicable, should be kept in ignorance as to the drug employed, so he can not get and use it of his own accord.—*T. Hilliard Wood, M. D., in Journal of Practical Medicine for March*, 1897.

